# Dangerous Miracle: The Astonishing Rise and Looming Disaster of Antibiotics

**DOI:** 10.3201/eid3206.260224

**Published:** 2026-06

**Authors:** John P. Mills

**Keywords:** antibiotics, antimicrobial resistance, penicillin, *Staphylococcus aureus*, streptomycin, isoniazid, books and media

It is easy to forget that the first antibiotic was discovered merely 100 years ago, yet its impact on medicine has been profound. In Dangerous Miracle: The Astonishing Rise and Looming Disaster of Antibiotics, University of Bristol evolutionary biologist Liam Shaw traces the rapid arc from discovery of the first antibacterial agents to the present-day crisis of ever-worsening antimicrobial resistance. Written for a general audience, the book weaves a highly readable journey through the science behind antibiotic development while situating their rise and decline within broader historical, political, and economic contexts.

Dangerous Miracle begins with the development of Prontosil, the first clinically useful sulfonamide, tracing its origins to the European dye industry. This account illustrates how advances in industrial chemistry unexpectedly intersected with medical therapeutics, ushering in the antimicrobial era. Shaw then turns to penicillin, describing Alexander Fleming’s observation of mold-mediated inhibition of *Staphylococcus aureus* and the herculean efforts required to translate this discovery into a widely available drug. He emphasizes that penicillin’s success depended on extensive multinational collaboration, particularly during World War II, when large-scale production became a strategic priority.

The author deftly chronicles the labor-intensive process of antibiotic discovery, including systematic screening of soil organisms for antimicrobial activity. He adds drama by highlighting disputes over scientific achievements and providing recognition for underappreciated contributors, such as Albert Schatz’s discovery of streptomycin. The discussion of streptomycin and isoniazid is enhanced by a personal narrative involving the author’s grandfather’s protracted bout with tuberculosis, which contextualizes the dramatic change these drugs brought to the treatment of a previously fatal disease.

The later portions of the book shift toward the structural and economic forces shaping contemporary antimicrobial development. Shaw argues that antibiotics are exploited in a manner similar to fossil fuels. Factors such as the consolidation of pharmaceutical research within large multinational companies and robust patent protection laws have contributed to a decline in antibiotic innovation. He situates those trends alongside the problems created by the emergence of first methicillin-resistant *Staphylococcus aureus* and then carbapenem-resistant Enterobacterales, framing resistance as both a microbiologic and systems-level problem.

In the final chapters, Dangerous Miracle examines the economic paradox of antibiotics: expensive to develop yet intended for short courses and judicious use, often constrained by antimicrobial stewardship programs. Limited space is devoted to potential solutions, but Shaw does advocate for several policy responses, including subscription-based reimbursement models that decouple profitability from sales volume and international public–private partnerships designed to support antimicrobial research and development. Although such proposals are discussed at a high level, the book offers limited evaluation of their feasibility or early outcomes.

Overall, Dangerous Miracle provides a balanced, chronological overview of the history of antibiotic discovery and the challenges posed by antimicrobial resistance. Its strengths lie in historical narrative and accessibility, rather than detailed policy analysis. This book will be of interest to clinicians, microbiologists, and public health experts seeking an overarching account of how one of medicine’s greatest successes has evolved into one of its most pressing global threats, and it serves as a call to action to reexamine how we invest in and regulate antimicrobials.

